# Untargeted Metabolomics Profiling of Bioactive Compounds under Varying Digestate Storage Conditions: Assessment of Antioxidant and Antifungal Activity

**DOI:** 10.3390/ijerph19084923

**Published:** 2022-04-18

**Authors:** Jiaxin Lu, Atif Muhmood, Panagiotis Tsapekos, Xian Cui, Yuwen Guo, Yi Zheng, Yizhan Qiu, Pan Wang, Lianhai Ren

**Affiliations:** 1 School of Ecology and Environment, Beijing Technology and Business University, Beijing 100048, China; lujiaxin@btbu.edu.cn (J.L.); guoyw11@163.com (Y.G.); zhengyi0805@126.com (Y.Z.); 2130051001@st.btbu.edu.cn (Y.Q.); 2 State Environmental Protection Key Laboratory of Food Chain Pollution Control, Beijing Technology and Business University, Beijing 100048, China; 3 Key Laboratory of Cleaner Production and Integrated Resource Utilization of China National Light Industry, Beijing Technology and Business University, Beijing 100048, China; 4 Institute of Soil Chemistry & Environmental Sciences, Ayub Agricultural Research Institute, Faisalabad 38000, Pakistan; atif_1534@yahoo.com; 5 Department of Chemical and Biochemical Engineering, Technical University of Denmark, 2800 Kongens Lyngby, Denmark; ptsa@kt.dtu.dk; 6 State Key Laboratory of Food Science and Technology, Engineering Research Center for Biomass Conversion, Nanchang University, Ministry of Education, Nanchang 330047, China; cuixian@ncu.edu.cn

**Keywords:** digestate storage, metabolite profiling, derivative pathways, antioxidant activity, antifungal activity

## Abstract

The rapid development of biogas plants in China has generated large quantities of digestate. The disparity between the continuity of biogas plant operation and the seasonality of digestate utilization has led to the need to store digestate. Therefore, untargeted profiling of bioactive compounds in the digestate stored under aerobic and anaerobic conditions was performed. The antioxidant and antifungal activity of digestate stored under varying conditions was likewise assessed. The results delineated that digestate storage under varying conditions brought about the degradation of organic acids, alkenes, aldehydes, alcohols, ketones, ethers, amino acids and their derivatives, and esters, leading to the stabilization of digestate components. Together, these new data revealed that digestate storage for up to 20 days under aerobic conditions promotes glycine, serine, and threonine degradation pathways and enhances biotin and vitamins production. In contrast, anaerobic storage enhances the taurine and hypotaurine metabolic pathways and increases the derivation of antimicrobial substances, such as indole alkaloids. Moreover, digestate storage under anaerobic conditions promotes antioxidant and antifungal activity more than storage under aerobic conditions. These findings can contribute to the future development of high-value agricultural products from digestate and the sustainability of biogas plants. Further studies are required for the untargeted metabolomic of digestate under storage to explore the underlying mechanisms of promoting disease resistance by the digestate upon land application.

## 1. Introduction

In recent decades, the demand for animal-derived food has risen dramatically due to continuing population growth and a shift in diet toward meat consumption [1]. Increased protein-based food consumption has resulted in rapid, intensive livestock farming industrialization [2]. This quick industrialization of animal husbandry has prompted the generation of huge amounts of animal manure, posing a serious threat to the soil and water environment [3,4]. The anaerobic digestion (AD) process and its mild operating conditions of complex organic carbon distinguish it from conventional energy technologies, making it a highly appealing and sustainable green energy technology for manure treatment, producing methane-rich biogas and nutrient-rich digestate [5,6]. Since it is high in readily available macro- and micronutrients, anaerobic digestate is a beneficial soil amendment [7].

Despite its well-known agronomic benefits, the use of digestate as an organic fertilizer or soil amendment in farmlands is dependent on plant and crop seasonality. Besides this, the oversupply and improper distribution of digestate, especially in the regions of intensive biogas plants and its application at an inappropriate time, enhance air and water pollution [8,9,10]. Consequently, before favorable agronomic applications in adjacent or remote farmlands, digestate had to be stored onsite for a certain period (up to 10 months) [11]. However, the storage and transport of digestate would bring about gaseous emissions and nutrient leaching, resulting in various environmental concerns, such as global warming and eutrophication [12,13].

Digestate is usually stored in open and closed reservoirs after solid–liquid separation. Digestate storage in open tanks leads to various problems, such as gaseous and odor emissions, the production of toxic compounds, and pathogen regrowth, thus limiting its potential fertilizing value [14]. Few studies have already been conducted for evaluating gaseous emission, antibiotics, and heavy metals removal from digestate during storage. For example, Vergote et al. [15] observed that nitrous oxide and methane emissions increased with digestate storage volume and temperature increase. In contrast, Mehta et al. [16] concluded that around 76% of doxycycline was removed with the storage of digestate under anaerobic conditions. Similarly, Li et al. (2018) reported an increase in organic matter, nutrients, and heavy metals during the storage of digestate openly under mesophilic conditions [17]. Studies on variations in the contents and type of bioactive compounds in the digestate during storage are scarce. Under anaerobic conditions, the degradation of organic compounds results in recalcitrant fractions, like biopolymers, steroids, and lignin [18]. Bioactive compounds, such as fatty acid derivatives, alkaloids, flavonoids, and terpenoids generated during the digestion of organic matter, are responsible for the disease resistance property of the digestate [19,20]. Thus, profiling bioactive compounds in the digestate during storage is necessary for evaluating antimicrobial and antioxidant activity and the underlying mechanism of promoting disease resistance by the digestate upon land application.

Therefore, to address this knowledge gap, the current study was undertaken for untargeted metabolomics profiling and the meta-analysis of biochemical changes that occur during digestate storage at different temperatures (4 °C, 20 °C, and 30 °C) under aerobic and anaerobic conditions based on the cloud-based mass spectrometric data analysis software (XCMS) integrated with the MetaboAnalyst program. Furthermore, the contents and type of bioactive compounds in the digestate preserved under aerobic and anaerobic conditions were compared. The antifungal and antioxidant activity of the digestate under varying storage conditions was also evaluated.

## 2. Materials and Methods

### 2.1. Experimental Material

Anaerobic digestate was collected from a continuous;y stirred tank reactor fed with swine manure at Donghuashan Biogas Plant (40.2° N, 116.9° E) in Beijing, China. Basic physicochemical characteristics of the digestate are given in Table 1.

### 2.2. Experimental Set-Up

To evaluate the variations in untargeted metabolomics profiling of bioactive compounds, about 2 L of digestate was placed in amber glass bottles and incubated for three months at 4 ± 1 °C, 20 ± 1 °C, and 30 ± 1 °C under aerobic and anaerobic conditions. An-4, Ae-4, An-20, Ae-20, An-30, and Ae-30 were the names given to the digestate held under aerobic and anaerobic conditions at the formerly mentioned temperatures. Each treatment was repeated three times, with digestate samples obtained weekly from each treatment.

### 2.3. Antioxidant Activity of Digestate

The antioxidant activity of digestate was determined by the DPPH (2,2′-diphenyl-1-picrylhydrazyl) method and then converted into the corresponding Trolox equivalents. Firstly, 7.856 mg of DPPH was weighed and dissolved in 200 mL of methanol to make a DPPH solution. After that, 1 mL of the digestate from different storage modes was mixed thoroughly with 4.5 mL of DPPH solution, left in the dark at room temperature for 30 min, and then the absorbance was measured at 517 nm (*A_test_*). Around 5.006 mg of Trolox was dissolved in distilled water to obtain a 200 μmol Trolox standard stock solution. A total of 1 mL of Trolox standard solution was then mixed with 4.5 mL DPPH solution and used as a control (*A_control_*). A mixture of 1 mL of methanol and 4.5 mL of DPPH solution was used as a blank (*A_blank_*) [21]. The antioxidant inhibition rate of the digestate stored under different conditions was calculated using the following equation:

$$Inhibition\;\left(\%\right)=\frac{A_{blank}-\left(A_{test}-A_{control}\right)}{A_{blank}}\times 100\%$$

A series of working standards from 20 to 60 μmol L^−1^ was prepared by diluting the Trolox standard stock solution using distilled water. To convert the DPHA radical scavenging rate into Trolox equivalents, a standard curve was drawn, with clearance (*x*) as the independent variable and Trolox equivalent (*y*) as the dependent variable.

The regression equation was as follows:

$$y=2.39x+1.65\;(R^{2}=0.9942)$$

where *x* is DPPH radical scavenging rate (%), and *y* is the Trolox equivalent (μmol/L).

### 2.4. Antifungal Activity of Digestate

The antifungal activity of digestate from different storage modes was measured against the pathogenic fungus (*Fusarium oxysporum*), which was procured from the Institute of Plant Protection, Chinese Academy of Agricultural Sciences, Beijing, China. The fungi were inoculated into a potato dextrose-agar (PDA) (Beijing Auboxing Biotechnology Co., Ltd., Beijing, China) medium on culture plates for 4 to 5 days for rejuvenation and then used as an inoculum. The PDA medium was sterilized by autoclaving at 121 °C for 20 min. When the autoclave temperature was reduced to 40–50 °C, the extracts (dichloromethane: methanol, 3:1) from the digestate in different storage modes were added to the melted PDA medium at a concentration of 5%. After thorough mixing, the medium was poured into petri dishes. The rejuvenated fungal colonies were taken out of the incubator for inoculation. Mycelial pads (5 mm diameter) were taken from the leading edge of actively growing colonies using stainless-steel forceps and put upside-down in the middle of the petri dish. The inoculated plates were then incubated at 25 ± 2 °C for one week. In addition, a mixture of pure extractant (Dichloromethane: Methanol = 3:1) and PDA was used as a blank control. The antifungal test was carried out in triplicate. The diameter of the colonies was measured every 24 h for one week using Vernier calipers. The relative inhibition growth (*RIG*) rate was calculated as described by Tao et al. [22].

$$\mathrm{RIG}=\frac{{(D}_{0}-\;D)}{D_{0}}\;\times \;100\%$$

*D*_0_ = colony diameter mixed with pure extractant (dichloromethane: methanol = 3:1) as the medium (cm), and *D* = colony diameter as a mixture of different storage modes of methane (cm) as a medium (cm).

### 2.5. Analytical Methods

#### 2.5.1. Sample Preparation for GC–MS

The digestate (20 mL) from each storage mode was blended with an equal amount of extractant (dichloromethane: methanol = 3:1) in a covered test tube. After that, 10 g of sodium chloride was added, and the mixture was agitated on an oscillator for one hour. An ultrasonic instrument was used to assist with the extraction process (KH300SP, 25 kHz, 300 W, Kunshan Ultrasonic Instruments Co., Ltd., Kunshan, China). The samples were first centrifuged for 30 min at 4032× *g*. After that, the supernatant layer was collected, and the rest was discarded. The extract obtained from the previous test was evaporated to 1.5 mL using a rotary evaporator (Heidolph Laborota 4001, Schwabach, Germany) at 40 °C and under a vacuum at 96 kPa.

#### 2.5.2. GC–MS Analysis

Digestate extract analysis was conducted using a GC–MS (Agilent 7890/5975, Santa Clara, CA, USA), equipped with a DB-5MS capillary column (30 m length, 0.25 mm inner diameter, 0.25 µm film thickness) and a quadrupole analyzer, operated in electron impact (70 eV) mode, with an *m*/*z* ranging from 35 to 600. The column was programmed so that the temperature increased at a rate of 5 °C min^−1^, from 60 °C to 180 °C; then, the rate was increased to 10 °C min^−1^ until the column reached 280 °C. The temperature was then maintained at 280 °C for 10 min. About 1.5 µL of the extract was then injected into the GC–MS using a micro-syringe, and scanning was performed for 50 min. After separation and detection, the compounds were eluted from the column and identified by comparing their mass spectra using the NIST08 data library [23].

#### 2.5.3. Data Processing and Statistical Analysis

The raw GC–MS data were uploaded to the XCMS online program for peak identification, peak filtering, and peak alignment to obtain a data matrix including the nucleus to mass ratio and retention time and peak area. The processed data were subjected to a multivariate statistical analysis using SIMCA-P (version 14.1; Umetrics, Umea, Sweden). An unsupervised multivariate pattern recognition method, namely a principal component analysis (PCA), was performed to examine the intrinsic variation in the data set. The supervised projection of orthogonal partial least squares discriminant analysis (OPLS-DA)) was performed for class discrimination. The quality of the OPLS-DA model was then assessed using 200 permutation tests. Variables were selected based on their effect on projection (VIP), reflecting the correlation between different storage conditions and the digestate composition.

To confirm the metabolic substances significant for digestate under varying storage conditions, all ions were sorted in descending order according to their VIP values. Potential metabolic markers were selected based on VIP values >1 and critical *p*-values from *t*-tests. The effect of aerobic or anaerobic storage on the most critical metabolites in the digestate was evaluated under varying storage temperatures, with a similarity index (SI) of >70% for the variables.

#### 2.5.4. Pathway Analysis

To discover the differential metabolites that significantly changed in the experimental groups, a pathway analysis was performed using MetaboAnalyst 4.0, a web-based metabolomics data analysis software. An enrichment analysis was performed based on the Kyoto Encyclopedia of Genes and Genomes (KEGG) database among these differential metabolites. Pathways with impact values > 0 and –log (*p*) > 2 (*p* < 0.01) were considered to be the most significant derivative pathways for the generation of metabolites.

## 3. Results and Discussion

### 3.1. Digestate Storage under Varying Conditions Changes Its Metabolites Profile

Based on the frameworks installed in the materials and methods and the multi-group analysis, the pre-processing of raw GC–MS data from digestate under varying storage conditions was conducted using the XCMS-Online software. XCMS-generated cloud plots allow an effective representation of GC–MS-based metabolomics data by providing information including the *p*-value, the directional fold change, the retention time, and the mass-to-charge ratio of metabolic features within a defined threshold [24,25]. The calibration procedure used for visualization and quality control, including an overlay of all chromatograms acquired before and after Rt. calibration, is shown in Figures S1 and S2. Based on the above pre-processing, around 1014 statistically significant features (*p* ≤ 0.05 *) were exported from the raw GC–MS data (Supplementary Materials). Moreover, the Non-Metric Multidimensional Scaling (NMDS) biplot provides a more straightforward visualization of authenticated samples, representing dissimilarities as a function of distance (Figure S3).

**Figure 1:**
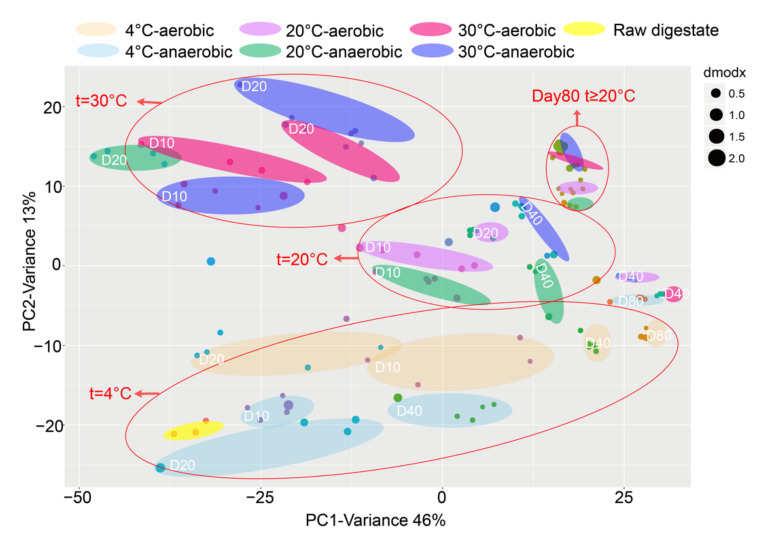
PCA distribution of digestate during different storage modes.

In addition, unsupervised principal component analysis (PCA) models were constructed on datasets after preliminary processing to get the intuitive metabolic distribution in the digestate under different storage modes (Figure 1). The PCA scores of PC1 and PC2 from the variable’s covariance matrix were 46% + 13%. The depicted clusters of samples in the same group proved the accuracy of the analytical methodology. A certain degree of separation was observed between the digestate samples from different storage modes. This proved that the differences between samples and sample sets were generated by actual biological differences, rather than instrumental effects. In addition, there was a clear separation among the digestate at different storage temperatures and durations. Moreover, with increased storage time, the location of samples in the PCA score plot moved slowly from left to right, indicating a gradual change in the composition of the digestate. Furthermore, the storage temperature strongly affected the distribution of plots. The effect of storage temperature on the composition of the digestate was explicitly shown by the slow movement of the samples from the bottom to the top of the PCA scoring plot. However, when the storage temperature was ≥20 °C, the samples aggregated as a cluster after a storage duration of >40 days, especially at 80 days. Therefore, within 40 days of storage, the metabolite spectrum of digestate was significantly affected by the storage temperature. The composition of the digestate tended to be consistent after 80 days of storage. In addition, the PCA plot showed a separation in the metabolite distribution patterns of the digestate stored under anaerobic and aerobic conditions at 20 °C, which illustrates that air conditions affected the chemical composition of digestate at 20 °C under the first 40 days of storage. The chemical compositions of digestate under storage at 4 °C for more than 40 days were independent of the stored oxygen content.

### 3.2. Digestate Storage under Varying Conditions Generates Different Types of Metabolites

**Figure 2:**
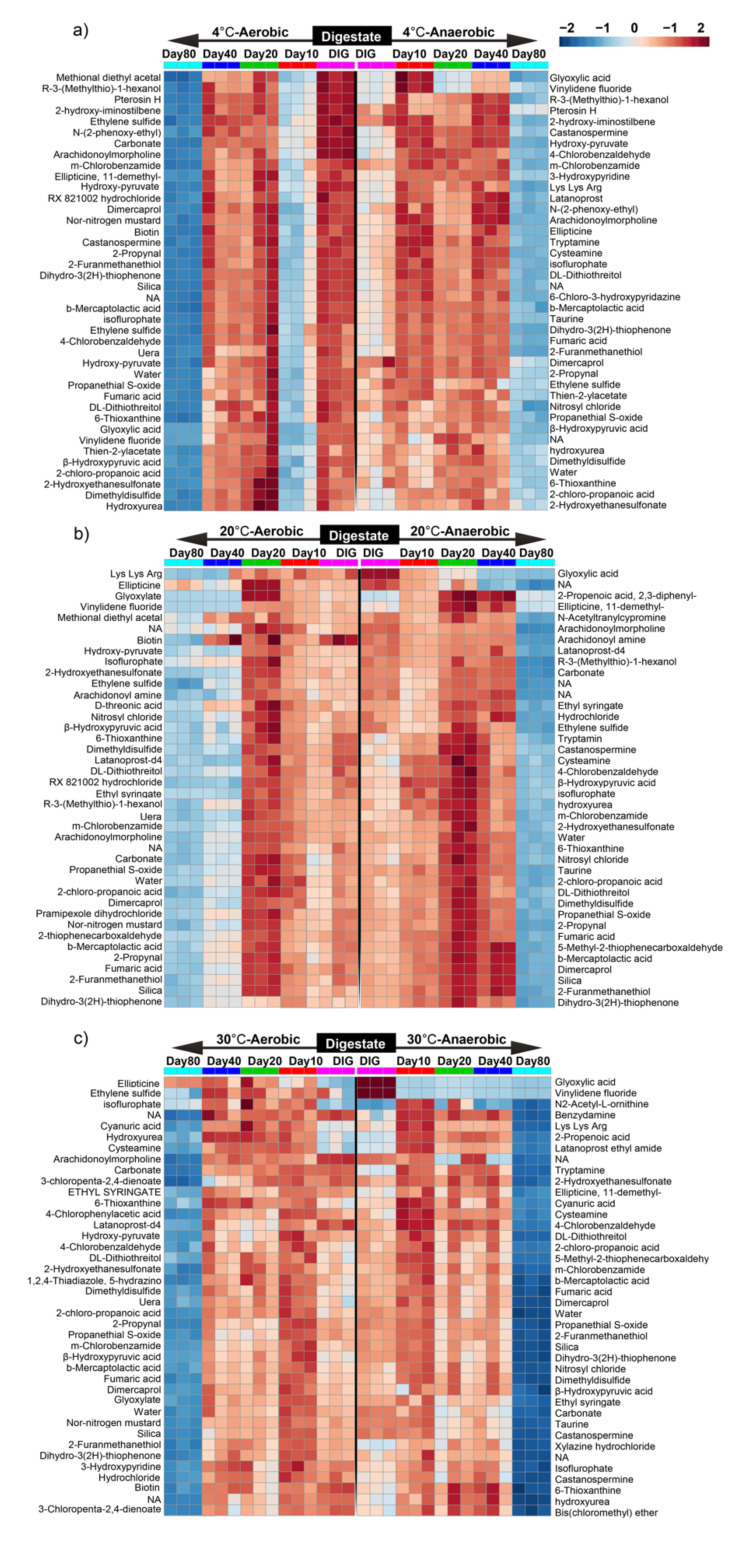
Heat map of the hierarchal clustering analysis of differential metabolites (*p* < 0.05) of digestate stored at (**a**) 4 °C aerobic versus 4 °C anaerobic, (**b**) 20 °C aerobic versus 20 °C anaerobic, and (**c**) 30 °C aerobic versus 30 °C anaerobic. Colored cells correspond to the concentration value (samples in column and compounds in row). The data presented were normalized and subject to a *t*-test/ANOVA, and features were standardized to autoscaling.

For each set of comparisons of original digestate and digestate from different storage modes (temperature, duration, and oxygen content), the Euclidean distance matrix of the differential metabolites was calculated. Moreover, the top 40 differential metabolites were clustered in an ultimately linked fashion and displayed on a heat map (Figure 2a–c). Significant metabolites, including volatile short-chain organic acids (2-chloro-propanoic acid, 2-Hydroxyethanesulfonate, b-Mercaptolactic acid, Carbonate, Fumaric acid, Glyoxylic acid, and β-Hydroxypyruvic acid), alkenes (Ethylene sulfide and Vinylidene fluoride), aldehydes (4-Chlorobenzaldehyde), alcohols (2-Furanmethanethiol, Dimercaprol, DL-Dithiothreitol, and R-3 (Methylthio)-1-hexanol), ketones (Dihydro-3(2H)-thiophenone, Pterosin H), ethers (Dimethyldisulfide, and Propanethial S-oxide), amino acids and their derivatives (6-Chloro-3- hydroxypyridazine, 6-Thioxanthine, Castanospermine, and hydroxyurea, *N*-(2-phenoxy-ethyl)), and esters (Arachidonoylmorpholine, 11-dimethyl-Ellipticine, Ethyl syringate, isoflurophate, and m-Chlorobenzamide Thien-2-ylacetate) were observed in digestate stored at 4 °C under aerobic and anaerobic conditions (Figure 2a).

When comparing aerobic and anaerobic storage conditions, the metabolites tanospermine, Methional diethyl acetyl, and Nor-nitrogen mustard were observed in digestate stored at 4 °C under aerobic conditions, while functional metabolites including 3-Hydroxypyridine and Latanoprost were observed in digestate stored under anaerobic conditions at 4 °C. In contrast, D-threonic acid, Lys Lys Arg, Nor-nitrogen mustard, and Vinylidene fluoride and 2,3-diphenyl-2-propenoic acid, Castanospermine, Cystanospermine, Cysteamine, 2-Hydroxyethanesulfonate, 4-Chlorophenylacetic acid, and *N*-Acetyltranylcypromine were observed for digestate stored at 20 °C under aerobic and anaerobic conditions, respectively (Figure 2b). Conversely, metabolites 3-Hydroxypyridine and Arachidonoylmorpholine were found in the digestate stored at 30 °C under aerobic conditions, and Castanospermine, 2,3-diphenyl-2-propenoic acid, 5-Methyl-2-thiophene carboxaldehyde, Benzydamine, Bis (chloromethyl) ether, and Glyoxylic acid were observed for digestate stored at the formerly mentioned temperature under anaerobic conditions (Figure 2c).

Furthermore, it was observed that the rate of degradation increased with incremented digestate storage duration, and the maximum degradation of components, including some organic acids, alkenes, aldehydes, alcohols, ketones, ethers, amino acids and their derivatives, and esters, occurred after 80 days of storage, resulting in the stabilization of the digestate composition. When comparing significant metabolites detected after 80 days of digestate storage at different temperatures, similar metabolites were observed for digestate storage at 4 °C and 30 °C under aerobic and anaerobic conditions; however, the metabolites 2,3-diphenyl-2-propenoic acid, Castanospermine, Taurine, Cysteamine, Tryptamine Cysteamine, 2-Hydroxyethanesulfonate, 4-Chlorophenylacetic acid, aand Acetyltranylcypromine were detected in the digestate stored at 20 °C under anaerobic conditions, while D-threonic acid, Hydroxy-pyruvate, Glyoxylate, Biotin, Uera, Lys Lys Arg, Nor-nitrogen mustard, and Vinylidene fluoride were detected in the digestate stored under aerobic conditions at the former temperature. These findings were consistent with the PCA analysis results, which represented a more significant contribution of oxygen content to the composition of the digestate at 20 °C storage.

### 3.3. Changes in the Metabolite’s Derivative Pathways under Varying Digestate Storage Conditions

**Figure 3:**
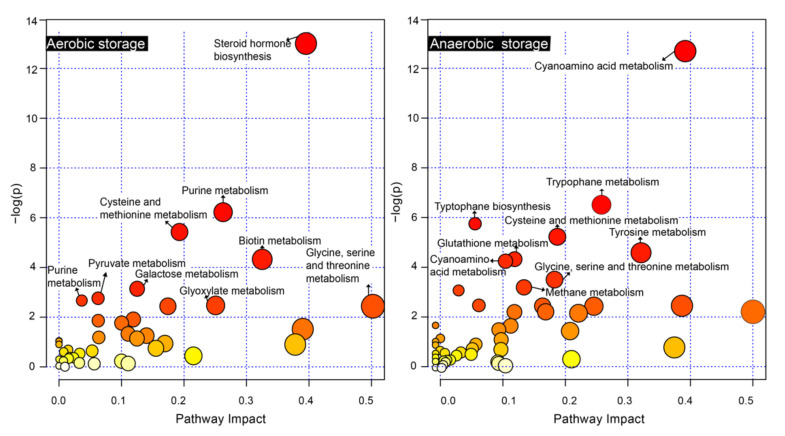
The metabolic pathways comparison of 20 °C anaerobic and aerobic storage modes for 20 days. (Each circle represents a metabolic pathway; the horizontal coordinate represents the impact value of that pathway, and the vertical coordinate represents the result of the enrichment analysis. The color of the circles changes from white to dark red as the −log(*p*) value increases, and the size of the circles is shown visually as the impact value increases from small to large, Impact > 0.05.).

To compare the metabolic changes in the digestate under aerobic and anaerobic storage conditions, an SMPDB enrichment analysis was performed by importing exclusive metabolites (*p* < 0.05) into MetaboAnalyst 5.0 (https://www.metaboanalyst.ca/faces/ModuleView.xhtml (accessed on 10 March 2022)) [26]. As shown in Table 2 and Table 3, 40 and 49 derivative pathways were observed for the digestate stored at 20 °C under aerobic and anaerobic conditions, respectively. Based on the analysis of p-values and impact values, the nine most significant metabolic pathways enriched in the digestate stored at 20 °C under anaerobic and aerobic conditions were selected (Figure 3). The specific metabolite derivative pathways in the digestate at 20 °C under aerobic conditions included steroid hormone biosynthesis; purine metabolism; cysteine and methionine metabolism; biotin metabolism; galactose metabolism; pyruvate metabolism; purine metabolism; glyoxylate metabolism; and glycine, serine, and threonine metabolism. In contrast, cyanoamino acid metabolism; tryptophan metabolism; tryptophan biosynthesis; cysteine and methionine metabolism; tyrosine metabolism; glutathione metabolism; cyanoamino acid metabolism; glycine, serine, and threonine metabolism; and methane metabolism were found to be responsible for metabolite generation in the digestate stored under anaerobic conditions at the formerly mentioned temperature.

Some of the metabolic pathways in the digestate were disturbed by different storage modes, leading to an impact on the active components therein, for example, a decrease in the metabolism of vitamin substances and an increase in the purine substance-derived pathway under aerobic storage conditions. An increase in indole acetic acid production by tryptophan metabolism occurred mainly in anaerobic storage.

#### 3.3.1. Aerobic Storage of Digestate Promotes the Biotin Metabolic Pathway for Vitamin Production

**Figure 4:**
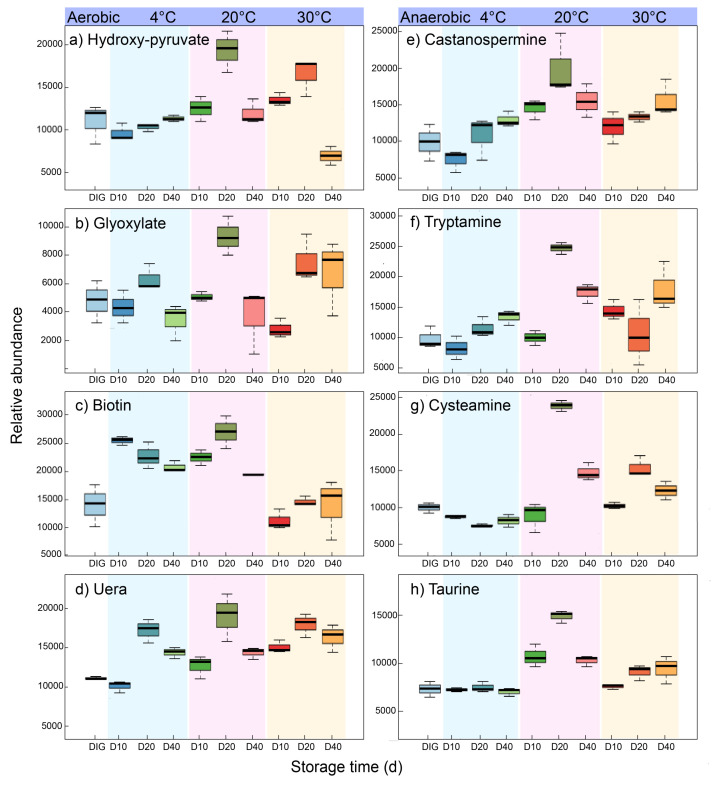
Box plots of the relative abundance of peak areas, representing the change tendency of eight bioactive compounds in the digestate under different storage modes. The a-axis represents the days of storage, and the y-axis represents the relative abundance of the bioactive compounds (mg/L).

**Figure 5:**
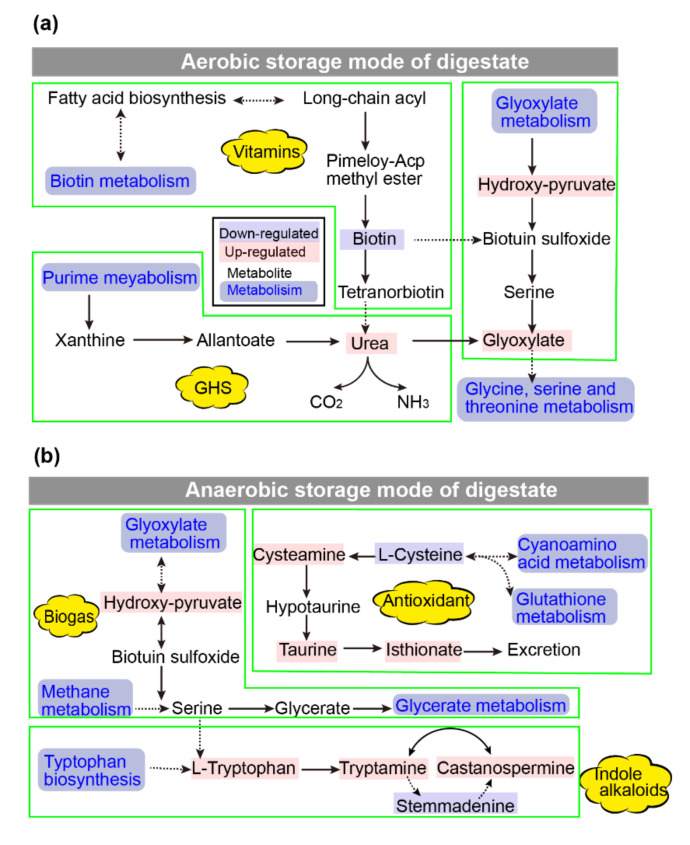
Schematic overview of the metabolic pathways in digestate stored under aerobic and anaerobic mode. (**a**) The main metabolic pathways of digestate in aerobic storage mode. (**b**) The main metabolic pathways of digestate in anaerobic storage mode.

Biotin is a water-soluble vitamin with antioxidant properties derived from the breakdown of volatile fatty acids, promoting plant root growth [27]. Biotin was detected in digestate stored under aerobic conditions (Figure 4c). Biotin metabolism starts with fatty acids biosynthesis and then methylates to form pimeloyl-ACP methyl ester and Pimeloyl-ACP. The products are then converted to biotin following a biotin bicyclic assembly step (Figure 5a). Biotin is catabolized by beta-oxidation of the valeric acid side chain or oxidation of sulfur in the heterocyclic ring [28]. At the same time, an inverse relationship was observed between storage temperature and biotin in aerobic storage. Therefore, the biotin metabolism in the digestate is boosted in aerobic storage, and the antioxidant properties of the digestate reduce with an incremented temperature and storage duration.

#### 3.3.2. The Aerobic Storage Mode Promoted Glycine, Serine, and Threonine Metabolism to Enhance the Antimicrobial Capacity

Digestate storage under aerobic conditions promoted the metabolism of glycine, serine, threonine, and purine metabolism. The transamination reaction between serine and glycine to form hydroxyl pyruvate and glycine is catalyzed by glyoxylate aminotransferase and requires oxygen’s involvement (Figure 4b). As the temperature increased, glyoxylate gradually increased with the transamination reaction between serine and glyoxylate. Glyoxylate has a significant antimicrobial ability and is a target product for developing many antimicrobial agents [29]. In addition, under the aerobic storage of digestate, the purine metabolic pathway was enhanced with further conversion to urea, explaining the reduced antioxidant properties under high-temperature aerobic storage conditions.

#### 3.3.3. Anaerobic Storage Enhances the Tryptophan and Lysine Metabolic Pathway to Produce Indole Alkaloids

The metabolism of tryptophan and lysine during anaerobic storage of the digestate resulted in the production of castanospermine and tryptamine (Figure 4e,f). It means the anaerobic environment facilitated the conversion of these readily catabolized amino acids to indole alkaloids. Moreover, the relative abundance of castanospermine and tryptamine gradually increased with an increasing temperature. The highest relative levels of castanospermine and tryptamine were observed at 20 °C and after 20 days of storage, with a relative abundance of 19,886 ± 4986 and 24,975 ± 1654, respectively, indicating an enhanced tryptophan metabolism. Castanospermine and Tryptamine are indolizidine alkaloids that have been identified in several synthetic antimicrobial agents as well as natural antibacterial ingredients. Castanospermine has been identified as having antibacterial, antifungal, and antitumour effects [30]. Tryptamine has antibacterial and anti-inflammatory effects [31,32].

#### 3.3.4. Anaerobic Storage Promotes the Taurine and Hypotaurine Metabolism Pathways to Produce Antioxidants

Taurine and hypotaurine metabolism pathways were detected in the digestate stored under anaerobic conditions, with the maximum relative abundance observed at 20 °C storage (Figure 4h and Figure 5b). Taurine in digestate is derived from cysteamine. The anaerobic condition activates anaerobic genes and promotes the production of oxygen-sensing enzymes, such as cysteamine oxygenase. These oxygen-sensing enzymes can control anoxia-dependent processes. For example, oxygen-sensing enzymes act on mutations of amino acids in their active sites, catalyze oxygen-dependent oxidation, and drive the addition of two oxygen atoms to free cysteamine to form taurine. They can also act on individual donors by incorporating molecular oxygen (oxygenase) to form taurine. Taurine can scavenge free radicals and attenuate lipid peroxidation; therefore, it can be used as an antioxidant to stabilize biofilms and applied in agriculture to enhance nutrient uptake by plants [33,34].

### 3.4. Antioxidant and Antifungal Properties of Digestate under Different Storage Modes

**Figure 6:**
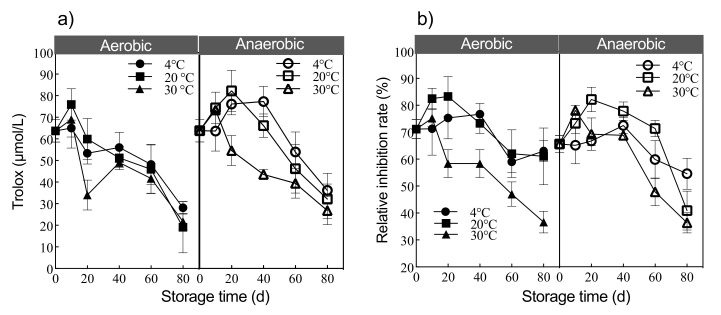
Antioxidant resistance and antifungal activity rate of digestate storage under varying conditions. (**a**) Antioxidant properties of digestate under different storage modes. (**b**) Antifungal properties of digestate under different storage modes.

To identify the effect of different storage modes on the antioxidant properties, digestate’s ability of digestate under varying storage conditions to scavenge DPPH radicals was determined (Figure 6). The antioxidant capacity of the digestate in both aerobic and anaerobic storage modes first increased in short-term storage (0–40 days) and then decreased with long-term storage (40–80 days). The maximum antioxidant capacity of digestate stored at 4 °C, 20 °C, and 30 °C under aerobic conditions was obtained up to 10 day’s storage and was approximately 65.03 ± 9.27, 76.00 ± 7.30, and 69.01 ± 8.20 μmol/L Trolox, respectively. The highest antioxidant capacity of the digestate stored at 4 °C, 20 °C, and 30 °C under anaerobic conditions was around 77.23 ± 7.04, 82.15 ± 9.5, and 73.27 ± 0.20 μmol/L Trolox, respectively. In comparison, the minimum antioxidant activity (<50 μmol/L Trolox) was obtained after 60 days of storage under different modes.

The maximum antifungal activity of digestate against *Fusarium oxysporum* was observed for digestate stored at 20 °C under aerobic and anaerobic conditions (83.3 ± 7.37% and 89.8 ± 5.13%, respectively) (Figure 6b). Conversely, the antifungal activity was minimum for the digestate stored at 4 °C under aerobic and anaerobic conditions (76.7 ± 4.04% and 78.9 ± 3.37%, respectively). Moreover, it was perceived that the antifungal activity decreased with an increase in storage duration. For low-temperature storage at 4 °C, the digestate could be stored for up to 40 days, regardless of oxygen content. Storage at 20 °C in an anaerobic state contributed to the best antioxidant and antifungal properties of the digestate. Storage of digestate at 30 °C for merely 10 days resulted in a significant reduction in antimicrobial activity. The short storage duration of the digestate helped to maintain the relative stability of the digestate, probably due to the conversion of proteins, amino acids, and sugars and fats into secondary derivatives in the digestate.

The increased antioxidant and antifungal activity in the digestate stored under anaerobic conditions was because of the accumulation of indolizidine alkaloids from ricin and tryptamine. The accumulation of cysteamine and taurine in the digestate stored under anaerobic conditions further promoted antioxidant and antifungal activity (Figure 4 and Figure 5). Conversely, the accumulation of glyoxylic acid in the digestate stored under aerobic conditions was responsible for the antioxidant activity of the digestate (Figure 4 and Figure 5). Contrasted with the current study, Ramli et al. (2021) evaluated extracts from a *Durio zibethinus* peel and seed for their antioxidant and antimicrobial activity [35]. They reported that the extract from peels contained higher contents of total phenolics and flavonoids than the seed. They further observed that both the peel and seed exhibited antioxidant activity.

## 4. Conclusions

Untargeted metabolomics profiling in digestate stored at varying temperatures (4 °C, 20 °C, and 30 °C) and time (0 to 80 days) under aerobic and anaerobic conditions was performed. The antioxidant and antifungal activity of digestate stored under varying conditions was also evaluated. The results showed that the degradation rate increased with an increase in storage duration. The maximum number of metabolites was detected after 80 days of storage, irrespective of the temperature and mode (aerobic and anaerobic). Moreover, around 49 metabolic pathways of metabolite generation were observed for digestate stored under anaerobic conditions, while 40 metabolic pathways were detected in digestate stored under aerobic conditions. The maximum antioxidant and antifungal activity (82.15 ± 9.5 μmol/L Trolox and 89.81 ± 5.13%, respectively) were recorded for digestate stored at 20 °C for 20 days under aerobic conditions. This study also showed that metabolomics can be an innovative and promising approach to elucidate the derivation pathways of metabolites and provide new perspectives for a further analysis of digestate under varying storage conditions. More studies are needed for exploring untargeted metabolomic profiling and the antifungal activity of digestate under varying storage conditions to evaluate its disease resistance potential upon land application.

## Figures and Tables

**Table 1:** Basic physicochemical characteristics of the digestate.

pH	COD (g/L)	DOC (mg/L)	TN (g/L)	NH_4_^+^ (g/L)	TS (g/L)	VS (g/L)
7.17 ± 0.06	4.43 ± 0.02	1.2 ± 0.04	3.09 ± 0.06	1.09 ± 0.08	45.4 ± 0.1	28.4 ± 0.1

**Table 2:** The derivative pathway of digestate under aerobic storage.

Metabolic Pathway	Total	Expected	Hits	Raw *p*	−log(*p*)	Holm Adjust	FDR	Impact
Steroid hormone biosynthesis	99	4.73	17	2.25 × 10^−6^	1.3 × 10^1^	1.8 × 10^−4^	1.8 × 10^−4^	0.4
Purine metabolism	20	0.96	5	1.99 × 10^−3^	6.22	1.57 × 10^−1^	7.95 × 10^−2^	0.26
Cysteine and methionine metabolism	56	2.68	8	4.41 × 10^−3^	5.42	3.44 × 10^−1^	1.18 × 10^−1^	0.19
Biotin metabolism	11	0.53	3	1.32 × 10^−2^	4.33	1	2.65 × 10^−1^	0.33
Galactose metabolism	41	1.96	5	4.32 × 10^−2^	3.14	1	6.92 × 10^−1^	0.13
Pyruvate metabolism	32	1.53	4	6.34 × 10^−2^	2.76	1	6.99 × 10^−1^	0.06
Purine metabolism	92	4.4	8	6.94 × 10^−2^	2.67	1	6.99 × 10^−1^	0.04
Glyoxylate metabolism	22	1.05	3	8.45 × 10^−2^	2.47	1	6.99 × 10^−1^	0.25
Glycine, serine, and threonine metabolism	50	2.39	5	8.74 × 10^−2^	2.44	1	6.99 × 10^−1^	0.5
Starch and sucrose metabolism	50	2.39	5	8.74 × 10^−2^	2.44	1	6.99 × 10^−1^	0.17
Limonene and pinene degradation	59	2.82	5	1.48 × 10^−1^	1.91	1	1	0.12
Pyrimidine metabolism	60	2.87	5	1.56 × 10^−1^	1.86	1	1	0.06
Arachidonic acid metabolism	62	2.96	5	1.72 × 10^−1^	1.76	1	1	0.1
Methane metabolism	34	1.62	3	2.2 × 10^−1^	1.52	1	1	0.39
Caffeine metabolism	21	1	2	2.65 × 10^−1^	1.33	1	1	0.11
Nitrogen metabolism	39	1.86	3	2.85 × 10^−1^	1.26	1	1	0.14
Arginine and proline metabolism	77	3.68	5	3.06 × 10^−1^	1.18	1	1	0.06
Thiamine metabolism	24	1.15	2	3.19 × 10^−1^	1.14	1	1	0.12
Nicotinate and nicotinamide metabolism	44	2.1	3	3.52 × 10^−1^	1.04	1	1	0
One carbon pool by folate	9	0.43	1	3.57 × 10^−1^	1.03	1	1	0
Lysine degradation	47	2.25	3	3.92 × 10^−1^	9.38 × 10^−1^	1	1	0.17
Selenoamino acid metabolism	48	2.29	3	4.05 × 10^−1^	9.05 × 10^−1^	1	1	0
alpha-Linolenic acid metabolism	29	1.39	2	4.07 × 10^−1^	8.99 × 10^−1^	1	1	0.38
Terpenoid backbone biosynthesis	33	1.58	2	4.73 × 10^−1^	7.48 × 10^−1^	1	1	0.16
Propanoate metabolism	35	1.67	2	5.05 × 10^−1^	6.84 × 10^−1^	1	1	0.01
Tryptophan metabolism	79	3.77	4	5.28 × 10^−1^	6.39 × 10^−1^	1	1	0.05
Glutathione metabolism	38	1.82	2	5.5 × 10^−1^	5.99 × 10^−1^	1	1	0.01
Sulfur metabolism	18	0.86	1	5.87 × 10^−1^	5.33 × 10^−1^	1	1	0.03
Ascorbate and aldarate metabolism	45	2.15	2	6.43 × 10^−1^	4.42 × 10^−1^	1	1	0.22
Fructose and mannose metabolism	48	2.29	2	6.78 × 10^−1^	3.89 × 10^−1^	1	1	0.02
Alanine aspartate and glutamate metabolism	24	1.15	1	6.93 × 10^−1^	3.67 × 10^−1^	1	1	0.02
Tyrosine metabolism	76	3.63	3	7.14 × 10^−1^	3.37 × 10^−1^	1	1	0.02
Pantothenate and CoA biosynthesis	27	1.29	1	7.35 × 10^−1^	3.07 × 10^−1^	1	1	0
Glycerolipid metabolism	32	1.53	1	7.93 × 10^−1^	2.31 × 10^−1^	1	1	0.01
Lysine biosynthesis	32	1.53	1	7.93 × 10^−1^	2.31 × 10^−1^	1	1	0.1
Glycerophospholipid metabolism	39	1.86	1	8.54 × 10^−1^	1.58 × 10^−1^	1	1	0.03
Folate biosynthesis	42	2.01	1	8.74 × 10^−1^	1.34 × 10^−1^	1	1	0.11
Aminoacyl-tRNA biosynthesis	75	3.58	2	8.83 × 10^−1^	1.25 × 10^−1^	1	1	0.06
Metabolism of xenobiotics by cytochrome	65	3.11	1	9.6 × 10^−1^	4.05 × 10^−2^	1	1	0
Porphyrin and chlorophyll metabolism	104	4.97	1	9.95 × 10^−1^	5.5 × 10^−3^	1	1	0.01

**Table 3:** The derivative pathway of digestate under anaerobic storage.

Metabolic Pathway	Total	Expected	Hits	Raw *p*	−log(*p*)	Holm Adjust	FDR	Impact
Cyanoamino acid metabolism	99	5.63	17	2.57 × 10^−5^	1.06 × 10^1^	2.06 × 10^−3^	2.06 × 10^−3^	0.40
Trypophane metabolism	20	1.14	5	4.30 × 10^−3^	5.45	3.40× 10^−1^	1.72 × 10^−1^	0.26
Typtophane biosynthesis	32	1.82	6	8.08 × 10^−3^	4.82	6.31 × 10^−1^	2.16 × 10^−1^	0.06
Cysteine and methionine metabolism	56	3.19	8	1.25 × 10^−2^	4.38	9.62 × 10^−1^	2.50 × 10^−1^	0.19
Tyrosine metabolism	11	0.63	3	2.12 × 10^−2^	3.85	1.00	3.22 × 10^−1^	0.33
Glutathione metabolism	41	2.33	6	2.64 × 10^−2^	3.64	1.00	3.22 × 10^−1^	0.13
Cyanoamino acid metabolism	21	1.20	4	2.82 × 10^−2^	3.57	1.00	3.22 × 10^−1^	0.11
Glycine, serine, and threonine metabolism	48	2.73	6	5.20 × 10^−2^	2.96	1.00	5.20 × 10^−1^	0.19
Methane metabolism	39	2.22	5	6.73 × 10^−2^	2.70	1.00	5.97 × 10^−1^	0.14
Purine metabolism	92	5.24	9	7.46 × 10^−2^	2.60	1.00	5.97 × 10^−1^	0.04
Pyrimidine metabolism	60	3.42	6	1.23 × 10^−1^	2.10	1.00	7.08 × 10^−1^	0.07
Galactose metabolism	34	1.94	4	1.24 × 10^−1^	2.08	1.00	7.08 × 10^−1^	0.39
Lysine degradation	47	2.68	5	1.26 × 10^−1^	2.07	1.00	7.08 × 10^−1^	0.17
Selenoamino acid metabolism	22	1.25	3	1.26 × 10^−1^	2.07	1.00	7.08 × 10^−1^	0.25
Starch and sucrose metabolism	50	2.85	5	1.52 × 10^−1^	1.88	1.00	7.08 × 10^−1^	0.17
Glyoxylate and dicarboxylate metabolism	50	2.85	5	1.52 × 10^−1^	1.88	1.00	7.08 × 10^−1^	0.50
Thiamine metabolism	24	1.37	3	1.53 × 10^−1^	1.88	1.00	7.08 × 10^−1^	0.12
Tryptophan metabolism	79	4.50	7	1.59 × 10^−1^	1.84	1.00	7.08 × 10^−1^	0.23
Nicotinate and nicotinamide metabolism	44	2.50	4	2.39 × 10^−1^	1.43	1.00	9.71 × 10^−1^	0.00
Limonene and pinene degradation	59	3.36	5	2.43 × 10^−1^	1.42	1.00	9.71 × 10^−1^	0.12
Arachidonic acid metabolism	62	3.53	5	2.76 × 10^−1^	1.29	1.00	1.00	0.10
Terpenoid backbone biosynthesis	33	1.88	3	2.89 × 10-^1^	1.24	1.00	1.00	0.21
Biotin metabolism	38	2.16	3	3.69 × 10^−1^	9.98 × 10^−1^	1.00	1.00	0.01
Glycerophospholipid metabolism	39	2.22	3	3.85 × 10^−1^	9.55 × 10^−1^	1.00	1.00	0.10
One carbon pool by folate	9	0.51	1	4.10 × 10^−1^	8.91 × 10^−1^	1.00	1.00	0.00
Arginine and proline metabolism	77	4.38	5	4.49 × 10^−1^	8.01 × 10^−1^	1.00	1.00	0.06
Phenylalanine, tyrosine, and tryptophan biosynthesis	27	1.54	2	4.61 × 10^−1^	7.75 × 10^−1^	1.00	1.00	0.00
alpha-Linolenic acid metabolism	29	1.65	2	4.98 × 10^−1^	6.96 × 10^−1^	1.00	1.00	0.38
Fructose and mannose metabolism	48	2.73	3	5.21 × 10^−1^	6.52 × 10^−1^	1.00	1.00	0.06
Taurine and hypotaurine metabolism	31	1.76	2	5.35 × 10^−1^	6.26 × 10^−1^	1.00	1.00	0.10
Glycerolipid metabolism	32	1.82	2	5.52 × 10^−1^	5.94 × 10^−1^	1.00	1.00	0.01
Pentose and glucuronate interconversions	53	3.02	3	5.90 × 10^−1^	5.28 × 10^−1^	1.00	1.00	0.04
Propanoate metabolism	35	1.99	2	6.02 × 10^−1^	5.08 × 10^−1^	1.00	1.00	0.01
Steroid hormone biosynthesis	16	0.91	1	6.10 × 10^−1^	4.95 × 10^−1^	1.00	1.00	0.00
Aminoacyl-tRNA biosynthesis	75	4.27	4	6.28 × 10^−1^	4.66 × 10^−1^	1.00	1.00	0.06
Sulfur metabolism	18	1.02	1	6.53 × 10^−1^	4.26 × 10^−1^	1.00	1.00	0.03
Citrate cycle (TCA cycle)	20	1.14	1	6.92 × 10^−1^	3.69 × 10^−1^	1.00	1.00	0.00
Ascorbate and aldarate metabolism	45	2.56	2	7.37 × 10^−1^	3.05 × 10^−1^	1.00	1.00	0.22
Alanine, aspartate, and glutamate metabolism	24	1.37	1	7.57 × 10^−1^	2.79 × 10^−1^	1.00	1.00	0.02
Primary bile acid biosynthesis	47	2.68	2	7.59 × 10^−1^	2.76 × 10^−1^	1.00	1.00	0.02
Sphingolipid metabolism	25	1.42	1	7.71 × 10^−1^	2.60 × 10^−1^	1.00	1.00	0.01
Pantothenate and CoA biosynthesis	27	1.54	1	7.96 × 10^−1^	2.28 × 10^−1^	1.00	1.00	0.00
beta-Alanine metabolism	28	1.59	1	8.08 × 10^−1^	2.13 × 10^−1^	1.00	1.00	0.10
Tyrosine metabolism	76	4.33	3	8.19 × 10^−1^	2.00 × 10^−1^	1.00	1.00	0.02
Lysine biosynthesis	32	1.82	1	8.49 × 10^−1^	1.64 × 10^−1^	1.00	1.00	0.10
Inositol phosphate metabolism	39	2.22	1	9.00 × 10^−1^	1.05 × 10^−1^	1.00	1.00	0.01
Folate biosynthesis	42	2.39	1	9.17 × 10^−1^	8.72 × 10^−2^	1.00	1.00	0.11
Metabolism of xenobiotics by cytochrome	65	3.70	1	9.79 × 10^−1^	2.12 × 10^−2^	1.00	1.00	0.00
Porphyrin and chlorophyll metabolism	104	5.92	2	9.85 × 10^−1^	1.50 × 10^−2^	1.00	1.00	0.01

## Data Availability

Not applicable.

## Supplementary Materials

The following supporting information can be downloaded at: https://www.mdpi.com/article/10.3390/ijerph19084923/s1, Figure S1: The calibration procedure used for visualization and quality control, including an overlay of all total ion chromatograms acquired before Rt. Calibration; Figure S2: The calibration procedure used for visualization and quality control, including an overlay of all total ion chromatograms acquired after Rt. Calibration; Figure S3: Non-Metric Multidimensional Scaling (NMDS) biplot.
