# Supplementary material for: Untargeted Metabolomics Profiling of Bioactive Compounds under Varying Digestate Storage Conditions: Assessment of Antioxidant and Antifungal Activity
Source: Int J Environ Res Public Health. 2022 Apr 18;19(8):4923. doi: 10.3390/ijerph19084923 (PMC9027003; doi:10.3390/ijerph19084923)
Supplement: Supplementary file 1 [file ijerph-19-04923-s001.zip › ijerph-1655463-supplementary.pdf]

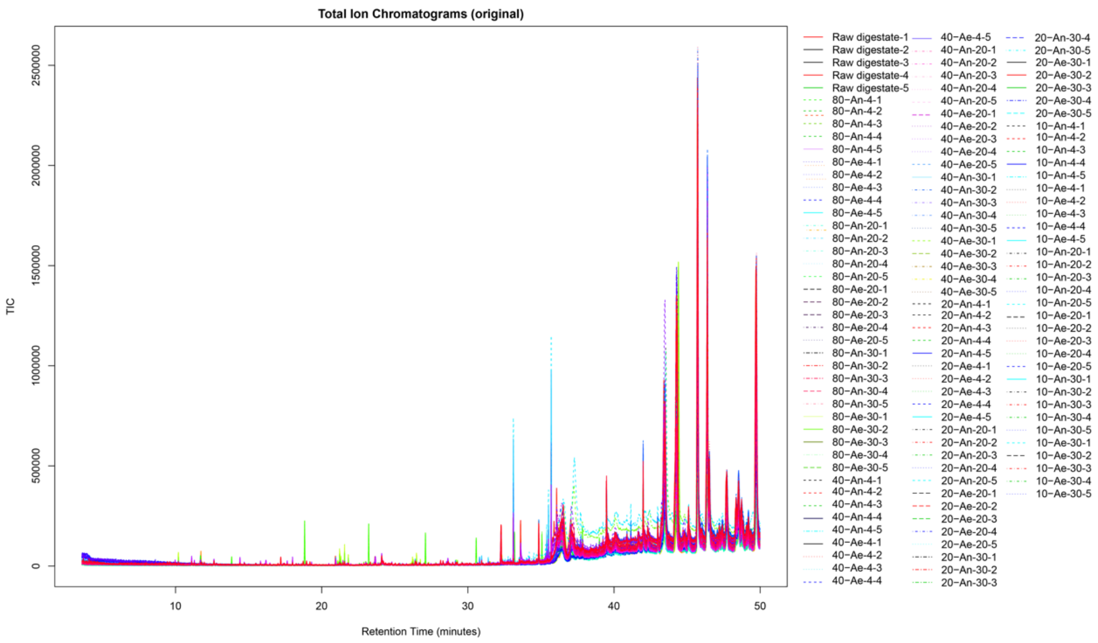

Figure S1. The calibration procedure used for visualization and quality control, including an overlay of all total ion chromatograms acquired before Rt. Calibration.

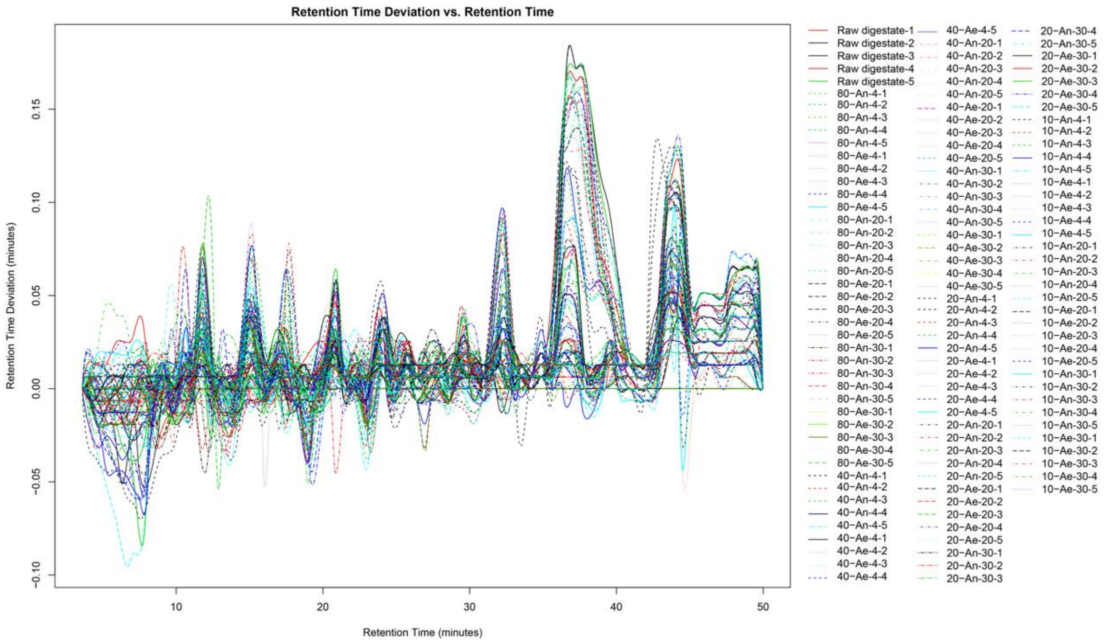

Figure S2. The calibration procedure used for visualization and quality control, including an overlay of all total ion chromatograms acquired after Rt. Calibration.

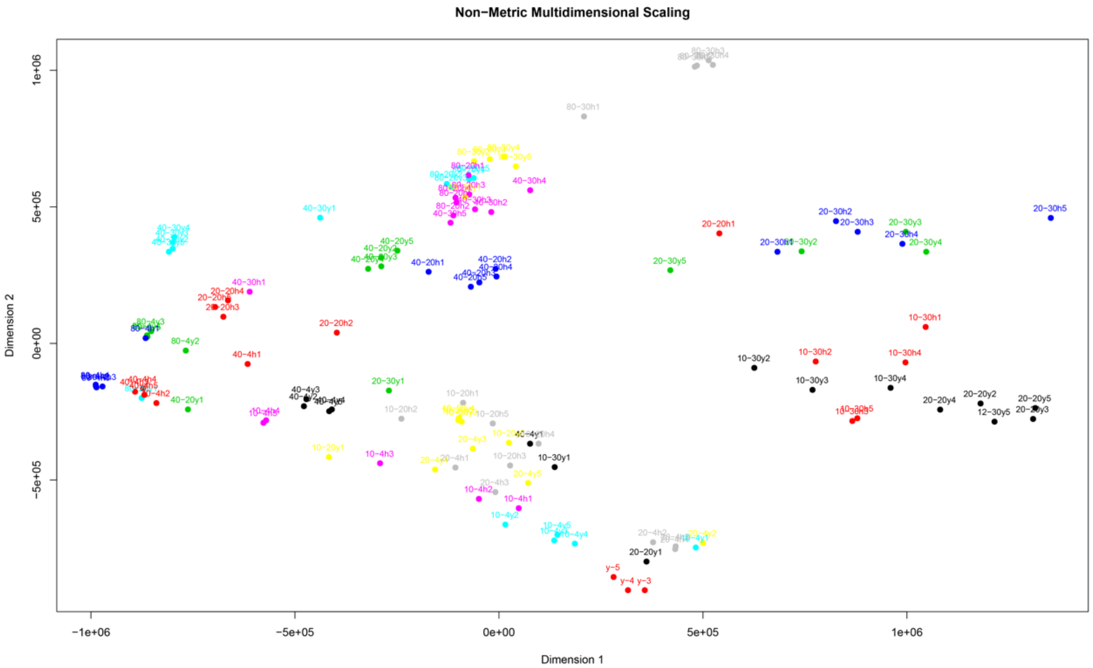

Figure S3. Non-Metric Multidimensional Scaling (NMDS) biplot.
